# Challenges for Opticians in Evaluating Small Pigmented Choroidal Lesions: Potential Support From the Mel*AI*noma Deep Learning Algorithm

**DOI:** 10.1167/tvst.14.6.29

**Published:** 2025-06-23

**Authors:** Shiva Sabazade, Marco A. Lumia Michalski, Sara Wittskog, Mats Holmström, Maria Nilsson, Gustav Stålhammar

**Affiliations:** 1 Ocular Oncology Service, St. Erik Eye Hospital, Stockholm, Sweden; 2 Department of Clinical Neuroscience, Division of Eye and Vision, Karolinska Institutet, Stockholm, Sweden; 3 Synologen AB, Stockholm, Sweden; 4 Expligence, Stockholm, Sweden; 5 St. Erik Ophthalmic Pathology Laboratory, St. Erik Eye Hospital, Stockholm, Sweden

**Keywords:** uveal melanoma, choroidal melanoma, opticians, optometrists, deep learning, artificial intelligence, Mel*AI*noma

## Abstract

**Purpose:**

To evaluate the diagnostic accuracy of Swedish opticians/optometrists when triaging small pigmented choroidal lesions and determine whether the Mel*AI*noma deep learning algorithm improves referral decisions.

**Methods:**

Twenty-nine opticians/optometrists graded 25 fundus photographs (5 melanomas, 20 nevi) with the Mushroom shape, Orange pigment, Large size, Enlargement, Subretinal fluid (MOLES) system and recorded referrals; 25 then used Mel*AI*noma. Predefined referral thresholds were MOLES ≥1 or ≥3 and Mel*AI*noma >63. Diagnostic test statistics—including sensitivity, specificity, positive (PPV) and negative (NPV) predictive values, accuracy, and standard deviation (SD)—plus odds of correct referral and decision curve net benefit were computed.

**Results:**

With MOLES ≥1, mean ± SD sensitivity was 98% ± 6%, specificity was 17% ± 16%, PPV was 23%, NPV was 99%, and accuracy was 33%. Raising the cutoff to MOLES ≥3 lowered sensitivity to 75% ± 29% but increased specificity to 53% ± 28%, PPV to 34%, NPV to 93%, and accuracy to 57%. Mel*AI*noma achieved 80% sensitivity, 90% specificity, PPV 67%, NPV 95%, and 88% accuracy. Algorithm guidance quadrupled the odds of correctly referring a melanoma, reduced false-positive referrals 10-fold, and provided net clinical benefit to unaided triage across all relevant threshold probabilities.

**Conclusions:**

Although the findings are based on a small image set, including only five melanomas, which limits generalizability, the results suggest that opticians and optometrists detect small choroidal melanomas with high sensitivity but limited specificity. Incorporating Mel*AI*noma at the predefined threshold reduced sensitivity, substantially improved specificity, and markedly reduced unnecessary referrals.

**Translational Relevance:**

Mel*AI*noma offers practical artificial intelligence support that can streamline community referrals and facilitate earlier treatment of uveal melanoma.

## Introduction

Choroidal nevi are relatively common, observed in approximately 4% to 7% of Caucasian adults.^1^^,^^2^ In contrast, uveal melanomas are rare but carry a risk of metastatic death, even in their smallest size category.^3^^–^^5^ Accurate differentiation between benign melanocytic lesions and true melanomas is crucial, as early and precise identification of malignant lesions likely offers the best opportunity for improving patient survival in this aggressive disease. The urgency of this issue is heightened by the increasing number of choroidal lesions detected incidentally through diabetic retinopathy screening programs, routine pre–cataract surgery evaluations, age-related macular degeneration treatment pathways, and the growing use of fundus photography among opticians and optometrists.^6^

Several established clinical methods help distinguish melanomas from nevi, primarily through the identification of clinical risk factors such as orange pigment and subretinal fluid.^7^^,^^8^ The likelihood of lesion growth is positively correlated with the number of these risk factors present.^9^ However, nevi may also grow, albeit slowly, complicating the distinction. Additionally, risk factors used to predict malignant transformation often overlap with diagnostic parameters for choroidal melanoma, which creates an inherent correlation regardless of the actual biological behavior of the lesion.^10^ Moreover, small lesions are rarely biopsied, making definitive pathological confirmation challenging. Small choroidal melanomas frequently resemble nevi at initial presentation, and lesions appearing indolent may start growing later. Thus, reliably distinguishing benign lesions from early malignant ones remains difficult even for experienced ocular oncologists, and this challenge is further amplified in health care systems where access to subspecialists is limited.

To address this issue, we recently developed and validated Mel*AI*noma, a deep learning algorithm designed to differentiate small choroidal melanomas from nevi.^11^ Mel*AI*noma, trained and tested on standard- and wide-field fundus photographs, demonstrated performance at least equivalent to that of experienced ocular oncologists. In the present study, we explore the experiences and challenges faced by opticians and optometrists in evaluating pigmented choroidal lesions. We assess their accuracy in distinguishing nevi from melanomas and evaluate whether the Mel*AI*noma algorithm can enhance their diagnostic performance and referral decisions.

## Methods

### Study Aims

The aim of this study was to evaluate the experience and challenges of opticians and optometrists in assessing pigmented lesions of the choroid and the scope for the Mel*AI*noma deep learning algorithm in helping them with these potential challenges.

### Design

This study involved Swedish opticians/optometrists who were invited to participate through Synologen AB, a nationwide ISO-certified Swedish member organization for opticians. The study was conducted in three steps: Participants first completed an initial questionnaire about their experience with pigmented choroidal lesions. They then assessed 25 images of such lesions, indicating the number of risk factors and whether referral to an ophthalmologist was warranted. Next, they tested the Mel*AI*noma algorithm, after which they completed a final questionnaire about their experience using the tool. All responses were anonymized to protect respondents’ privacy, and the authors did not have access to identifying information.

The study protocol was approved by the Swedish Ethical Review Authority (reference 2024-02620-02) and adhered to the tenets of the Declaration of Helsinki. The requirement for informed consent was waived because of the study's retrospective nature, relying solely on previously collected data, including clinical records and deidentified images. This research did not involve any new treatments, interventions, tests, analysis of biological samples, or collection of additional sensitive information.

### Optician and Optometrist Difference

In Sweden, an optician is a licensed health care professional with a 3-year university degree in optometry. Opticians perform routine vision examinations, prescribe and dispense eyeglasses and contact lenses, and refer patients for medical evaluation when necessary. Ophthalmologists generally do not participate in these tasks. An optometrist is an optician who has received additional formal training in ocular pharmacology, eye diseases, pediatric vision, and related areas, recognized by the National Board of Health and Welfare. This extended education grants the optometrist authority to prescribe and handle certain diagnostic drugs, allowing for a broader scope of practice in diagnosing and managing more complex ocular conditions. Both opticians and optometrists may operate within the Swedish health care system, adhering to regulations to ensure patient safety and uphold professional standards.

### Diagnostic Procedure

The 25 lesions evaluated by opticians/optometrists and the Mel*AI*noma algorithm had been diagnosed as either small choroidal melanomas or nevi during an in-person visit at the Ocular Oncology Service, St. Erik Eye Hospital. Our diagnostic protocol includes a review of each patient's medical history (prior diagnoses, medication regimens, and past ocular examinations) and a battery of assessments: best-corrected visual acuity and intraocular pressure measurement, wide-field or standard-field fundus photography with autofluorescence, optical coherence tomography, slit-lamp biomicroscopy, and A- and B-scan ultrasonography. In most cases, these evaluations allow for a definitive diagnosis of melanoma or nevus. If findings remain inconclusive, a transvitreal or transscleral biopsy is performed, as previously described.^12^^,^^13^ Consequently, all patients in this study underwent a comprehensive multimodal examination by an ocular oncologist, and anonymized standard-field photographs from these initial visits were used in our analyses. To be retained in the nevus group, a lesion had to be followed for >5 years after the initial visit without subsequent reclassification as melanoma. This requirement ensured that lesions labeled as nevi were not merely early melanomas that had yet to develop characteristic malignant features—changes likely to become evident within a few years. This rigorously defined benign reference set was essential for developing Mel*AI*noma, which is intended to separate small melanomas from their benign counterparts. Distinguishing large melanomas from nevi is comparatively straightforward.

### Instructions to Opticians/Optometrists

In addition to completing a questionnaire about their experience with pigmented choroidal lesions—including but not limited to age, sex, professional background, years in practice, approximate number of customers with such lesions encountered each year, and available imaging equipment—the opticians/optometrists were asked to assess the 25 images of pigmented choroidal lesions described in the previous section. They were instructed to assess each lesion using the Mushroom shape, Orange pigment, Large size, Enlargement, and Subretinal fluid (MOLES) criteria, and they received detailed instructions explaining the criteria, including definitions of each criterion and how points should be assigned.^8^ MOLES assigns a score of 0, 1, or 2 for Mushroom shape, Orange pigment, Large size, Enlarging tumor, and Subretinal fluid, reflecting their absence, borderline presence, or presence. Scores of 0, 1, 2, or >2 correspond to common nevi, low-risk nevi, high-risk nevi, and probable melanoma, respectively.

Next, they were granted access to a web-based version of the Mel*AI*noma algorithm. Finally, they completed a second questionnaire regarding their experience with the algorithm: whether they felt it would be useful in their practice, whether it could improve lesion assessments and the information provided to customers, and whether it would reduce the time required for assessments.

Referral criteria for pigmented lesions from opticians/optometrists to ophthalmologists are provided by the Swedish Association of Optometry. According to these written guidelines, a customer presenting with a pigmented lesion that has a MOLES score of ≥1 at the first evaluation should be referred immediately. If the MOLES score is 0, the lesion should be reexamined after 6 months; if it remains 0 at that time, the customer should be monitored yearly for 5 years and then every other year for life. If the MOLES score ever reaches ≥1, a referral is indicated. The guidelines also note that artificial intelligence (AI)–based systems are under development and are expected to provide support for opticians/optometrists and nonspecialized ophthalmologists. These guidelines were distributed to the participating opticians/optometrists prior to the study and were also reviewed verbally in an introductory meeting.

### Mel*AI*noma

Mel*AI*noma is a two-stage deep learning pipeline trained on standard- and wide-field fundus photographs, as described previously.^11^ Briefly, each image is resized to 1024 × 1536 pixels, brightness-normalized, and passed to a U-net with three down-sampling blocks that first segment the lesion and then classify a 488 × 488-pixel crop centered on the segmentation centroid. During training, random rotations and brightness-jitter augmentation are applied, categorical cross-entropy optimizes both networks, and the checkpoint with the highest validation area under the curve (AUC) is retained. Rank-ordered pixel-level melanoma probabilities are then processed by a shallow random forest ensemble with a 10:1 weighting for melanomas to minimize false-negative errors. An operating threshold of >63 was selected during development to balance sensitivity and specificity (although this threshold can be adjusted to prioritize sensitivity or specificity). The algorithm outputs a lesion-level melanoma probability score ranging from 0 (lowest likelihood) to 100 (highest likelihood).

### Statistical Analyses

Statistical significance was defined as two‐sided *P* < 0.05. Bonferroni correction was applied to all reported *P* values to account for multiple comparisons. For the assessment of diagnostic performance at predefined cutoffs (MOLES ≥1 and ≥3; Mel*AI*noma >63), sensitivity, specificity, positive predictive value (PPV), negative predictive value (NPV), and accuracy were calculated for each rater. Differences in referral proportions between melanomas and nevi were evaluated with two‐sample tests for equality of proportions (χ^2^ tests), and the associations between lesion type and referral decision were further examined by logistic regression analyses (binomial family, logit link), with nevi as the reference category. Receiver operating characteristic (ROC) analyses compared individual rater performance curves and the Mel*AI*noma algorithm. The statistical significance of AUC differences was assessed by DeLong's paired nonparametric test for correlated ROC curves. As a secondary distributional check, a paired Wilcoxon signed-rank test was performed on the 29 human AUCs versus the Mel*AI*noma AUC. Net clinical benefit across threshold probabilities (10%–90%) was estimated by decision curve analysis, defining net benefit (NB) as

$$\begin{array}{c} NB=\frac{TP}{n}-\frac{FP}{n}\times \frac{p_{t}}{1-p_{t}} \end{array}$$

where *TP* is the number of true positives (melanomas correctly referred), *FP* is the number of false positives (nevi incorrectly referred), *n* is the total number of lesions, and *p_t_* is the chosen threshold probability. All statistical analyses were conducted using R (version 4.4.3; R Foundation for Statistical Computing, Vienna, Austria) with the pROC, dplyr, and tidyr packages and with SPSS (version 29.0.2.0; IBM, Armonk, NY, USA).

## Results

### Descriptive Statistics

**Figure 1.**
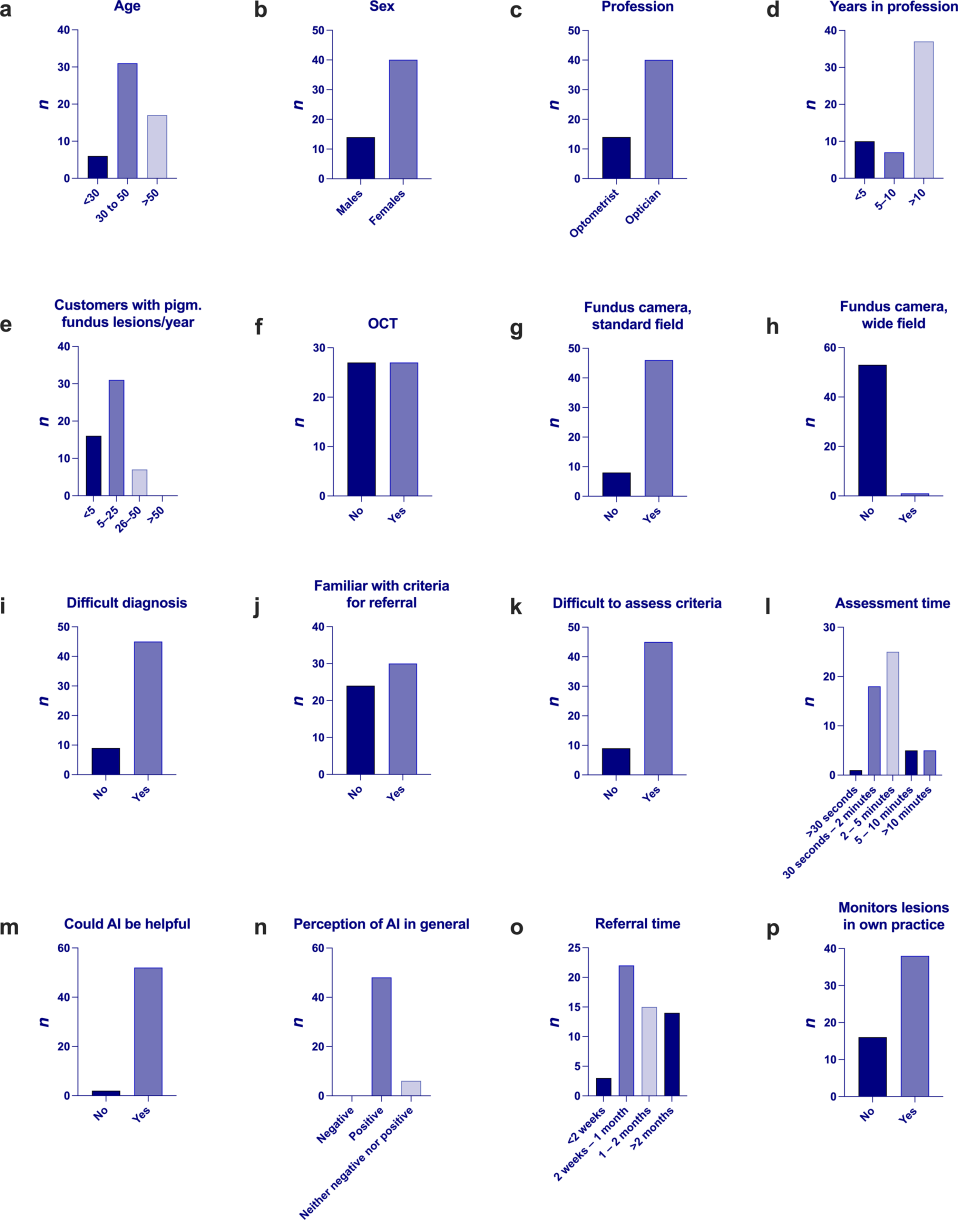
Opticians’ and optometrists’ experience with pigmented fundus lesions. Questions and response alternatives (with response alternatives in parentheses): (**a**) age (<30 years, 30 to 50 years, >50 years); (**b**) sex (man, woman, other/prefer not to say); (**c**) professional title (optician, optometrist, optical assistant, other); (**d**) years of professional experience (post-education) (<5 years, 5 to 10 years, >10 years); (**e**) annual number of customers with pigmented fundus lesions (include all individuals regardless of visit reason; estimate the number of individuals, not the number of lesions or visits) (<5, 5 to 25, 26 to 50, >50); (**f**) type of imaging equipment available in your store or practice (multiple responses possible) (OCT [optical coherence tomography], (**g**) conventional fundus camera [non–wide-field, e.g., Canon], (**h**) wide-field camera [e.g., Optos, Zeiss Clarus]); (**i**) do you think it is difficult to assess if a pigmented fundus lesion is benign or malignant (easy to assess, difficult to assess); (**j**) are you well acquainted with the criteria for referring lesions to an ophthalmologist (yes, no); (**k**) do you find it difficult to assess if these criteria are met (yes, no); (**l**) approximately how long does it usually take to assess a pigmented fundus lesion (exclude the time needed for image capture and patient communication; include time if you consult a colleague) (<30 seconds, 30 seconds to 2 minutes, 2 to 5 minutes, 5 to 10 minutes, >10 minutes); (**m**) do you believe that a deep learning–based tool (a form of artificial intelligence) could be helpful in these assessments of lesion risk or in deciding which lesions should be referred (yes, no); (**n**) are you generally positive or negative toward various artificial intelligence solutions for specific medical tasks (e.g., detecting tumors in mammograms, assessing skin lesions, etc.) (positive, negative, neither); (**o**) if a referral is sent to an ophthalmologist due to a pigmented fundus lesion, approximately how long does it take before the patient is scheduled for an examination (<2 weeks, 2 weeks to one month, 1 to 2 months, >2 months); and (**p**) do you currently follow up with a customer with a pigmented fundus lesion (for example, after an ophthalmologist has responded to your referral and advised you to continue monitoring) (yes, no).

A total of 54, 29, and 25 opticians/optometrists from 17 optician shops in 17 Swedish cities or towns participated in the first (questionnaire), second (fundus photo assessment), and third (testing of Mel*AI*noma) parts of this study, respectively. Of the 54 respondents to the first questionnaire, 40 (74%) were female, and 37 (69%) had more than 10 years of experience in the profession. Most (46 respondents, 85%) had access to a fundus camera in their setting. Specifically, 27 respondents (50%) had only a conventional (non–wide-field) fundus camera, 19 (35%) had both a conventional fundus camera and optical coherence tomography (OCT), 7 (13%) had only OCT, and 1 (2%) had both a wide-field fundus camera and OCT. Forty-five respondents (83%) reported difficulties determining whether the criteria for referral to an ophthalmologist were met. Thirty-five respondents (65%) indicated that assessing a pigmented lesion typically took them 2 minutes or longer, and 38 (70%) saw five or more unique customers with pigmented lesions each year. A complete breakdown of questionnaire responses is provided in Figure 1.

### Sensitivity, Specificity, and Predictive Values

The characteristics of the 25 patients whose pigmented lesions were included for assessment by opticians/optometrists are shown in Table 1. Notably, the median MOLES score, as determined by ocular oncologists at St. Erik Eye Hospital, was 3 (range, 0–4) for the small melanomas and 0 (range, 0–2) for the nevi; the number of risk factors according to the TFSOM-UHHD mnemonic (thickness >2 mm, presence of subretinal fluid, symptoms, orange pigment, tumor margin within 3 mm of the optic disc, and ultrasonographic hollowness) was 3 (range, 2–4) and 1 (range, 0–2), respectively.

**Table 1.** Baseline Characteristics of Patients With Pigmented Choroidal Lesions (*n* = 25)

Characteristic	Overall (*n* = 25)	Nevus (*n* = 20)	Melanoma (*n* = 5)
Age, years, mean ± SD (range)	61 ± 15 (34–85)	59 ± 16 (34–84)	72 ± 9 (61–85)
Sex, female, *n* (%)	15 (60)	15 (75)	3 (60)
MOLES score, median (range)^a^	0 (0–4)	0 (0–2)	3 (0–4)
TFSOM-UHHD risk factors, median *n* (range)^a^	1 (0–4)	1 (0–2)	3 (2–4)
AJCC T-category, *n* (%)			
T0	20 (80)	20 (100)	0 (0)
T1a	5 (20)	0 (0)	5 (100)
Treatment, *n* (%)			
None	20 (80)	20 (100)	0 (0)
Brachytherapy	5 (20)	0 (0)	5 (100)
Other	0 (0)	0 (0)	0 (0)
Vital status at data collection, *n* (%)			
Alive	20 (80)	17 (85)	3 (60)
Metastatic death	5 (20)	3 (15)	2 (40)
Other death	0 (0)	0 (0)	0 (0)

The MOLES score is based on the presence of five features—Mushroom shape, Orange pigment, Large size, Enlargement, and Subretinal fluid—with a score of 0, 1, or 2 assigned for each feature.

The TFSOM-UHHD risk factors include thickness >2 mm, presence of subretinal fluid, symptoms, orange pigment, tumor margin within 3 mm of the optic disc, and ultrasonographic hollowness. AJCC, American Joint Committee on Cancer.

a Assessed by an ocular oncologist.

The opticians/optometrists were blinded to the gold-standard diagnoses, as determined in multimodal examinations by ocular oncologists. A total of 25 images (5 melanomas and 20 nevi) were first evaluated using predefined referral thresholds. For the optometrists/opticians, performance metrics were computed for each of the 29 raters at two referral cutoffs, and the mean ± standard deviation (SD) values were then determined.

At a cutoff of MOLES ≥1, the mean sensitivity was 98% ± 6%, with a very low mean specificity of 17% ± 16%. This yielded a mean PPV of 23% ± 4% and a mean NPV of 99% ± 4%, resulting in an accuracy of 33%. When the cutoff was increased to MOLES ≥3, the mean sensitivity decreased to 75% ± 29%, while the mean specificity improved to 53% ± 28%. Under this threshold, the mean PPV rose to 34% ± 16%, the mean NPV decreased to 93% ± 7%, and the accuracy increased to 57%.

When Mel*AI*noma was used to score the same photos, the predefined referral cutoff of >63 produced a sensitivity of 80%, a specificity of 90%, a PPV of 67%, an NPV of 95%, and an accuracy of 88% (Table 2).

**Table 2.** Diagnostic Performance Metrics

Method	Cutoff	Sensitivity (%; Mean ± SD)	Specificity (%; Mean ± SD)	Accuracy (%)	PPV (%; Mean ± SD)	NPV (%; Mean ± SD)
Optometrists/opticians (MOLES scores)	≥1	98 ± 6	17 ± 16	33	23 ± 4	99 ± 4
Optometrists/opticians (MOLES scores)	≥3	75 ± 29	53 ± 28	57	34 ± 16	93 ± 7
Mel*AI*noma score^a^	>63	80	90	88	67	95

Values for the optometrist/optician scores represent the mean ± SD across 29 raters.

a No SD is reported for the Mel*AI*noma score because the algorithm produces an identical score with each image analysis, in contrast to the variability seen among human raters.

### Referral Decision Analysis

**Figure 2.**
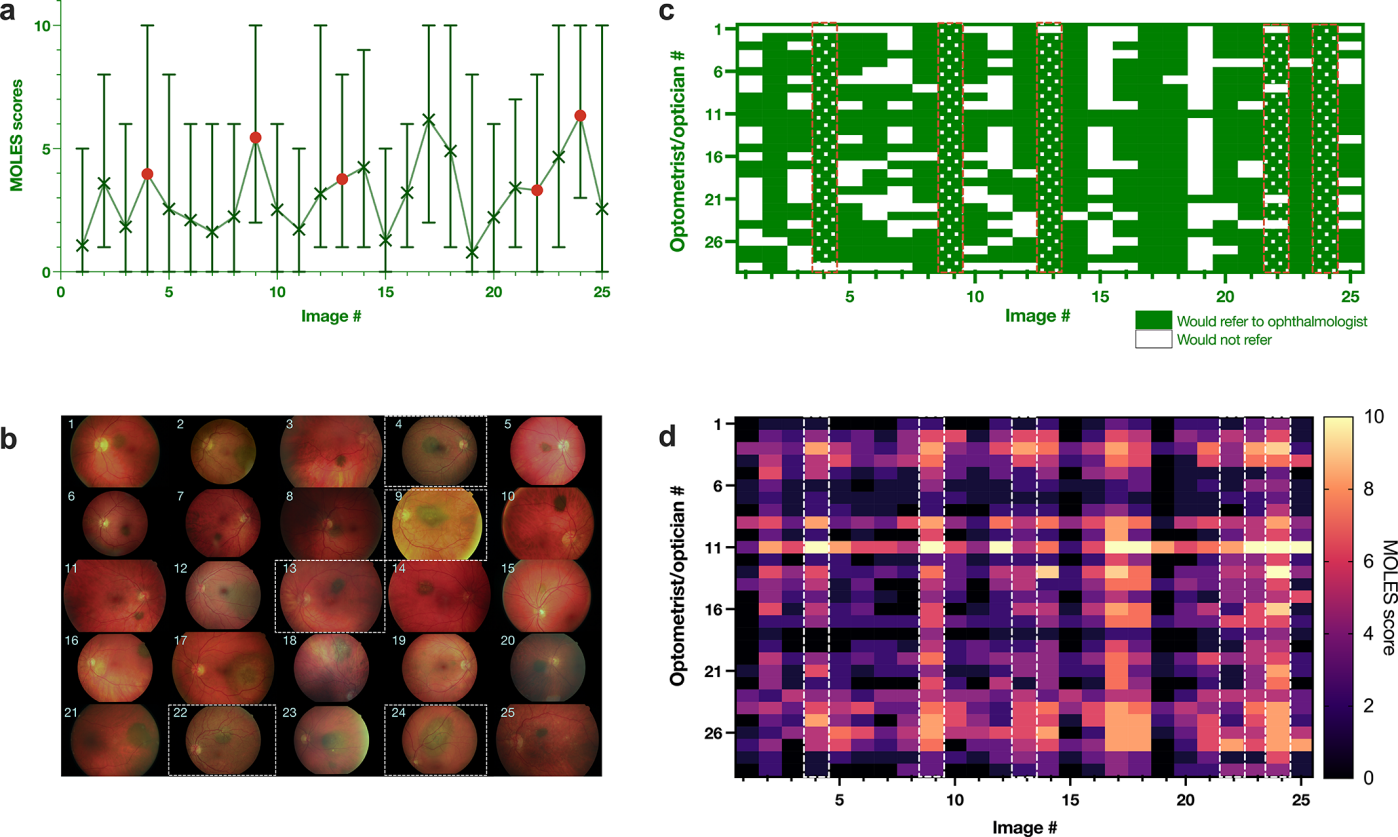
Opticians’ and optometrists’ MOLES scores and referral decisions for 25 pigmented fundus lesions. (**a**) Mean MOLES score per lesion (*n* = 29 raters), with *error bars* indicating the full range (minimum to maximum) of scores. *Green crosses* mark the mean score for lesions classified as nevi and *red dots* mark the mean score for lesions classified as melanoma. (**b**) The 25 fundus images; *white dashed boxes* identify the five melanomas confirmed by ocular oncologists and >5-year follow-up. (**c**) Heatmap of referral decisions: each row represents one optician/optometrist (*n* = 29) and each column one lesion (*n* = 25); *green cells* indicate “would refer” and *white cells* “would not refer.” (**d**) Heatmap of individual MOLES scores assigned by each rater to each lesion.

When the 25 lesions were evaluated by 29 opticians/optometrists, lesions diagnosed by ocular oncologists as melanomas received 138 referrals out of 145 individual decisions (95%), whereas lesions diagnosed as nevi received 397 referrals out of 580 decisions (68%, Fig. 2). The difference in referral proportions between lesions classified as melanomas and those classified as nevi was 27% (95% confidence interval [CI], 21%–32%). A two‐sample test for equality of proportions yielded χ^2^ = 42 and Bonferroni-corrected *P* = 0.005. Logistic regression analysis further confirmed these findings. Using nevi as the reference, the log odds of referral for lesions diagnosed as melanomas was significantly increased (β = 2.2, standard error [SE] = 0.4, *z* = 5.6, Bonferroni-corrected *P =* 0.005).

If the opticians/optometrists had based their referral decisions on the Mel*AI*noma score instead, applying a predefined referral threshold of >63, 4 of 5 melanomas would have been referred (80% referral rate) compared with 2 of 20 nevi (10% referral rate). Despite the small numbers, the two‐sample test for equality of proportions indicated a statistically significant difference (χ^2^ = 7.3, Bonferroni-corrected *P* = 0.03; 95% CI, 20%–100%). Logistic regression for the Mel*A*Inoma‐based decisions yielded a coefficient of β = 3.6 (SE = 1.3, *z* = 2.7, Bonferroni-corrected *P* = 0.03). Exponentiating these logistic regression coefficients gives an odds ratio of approximately 9.0 for opticians/optometrists’ decisions and 36.6 for Mel*AI*noma‐based decisions. Thus, the relative odds of referral for melanomas when using the Mel*AI*noma score were 4.1 times higher than those based on opticians/optometrists’ decisions.

### Decision Curve Analysis

Decision curve analysis was performed to compare the net clinical benefit of opticians/optometrists versus Mel*AI*noma across a range of threshold probabilities. For opticians/optometrists, the predicted referral probability for each lesion was defined as the proportion of “would refer” votes. In contrast, Mel*AI*noma will always yield the same score for the same image, with no variability. Table 3 shows the NB at threshold probabilities ranging from 10% to 90%. The threshold represents the minimum probability of a lesion being a melanoma at which referral is recommended, and NB is defined as the proportion of true positives minus the weighted proportion of false positives, where the weighting reflects the odds corresponding to the threshold probability. For example, at a threshold of 30%, opticians/optometrists yielded an NB of −0.1, whereas Mel*AI*noma achieved an NB of 0.1. Across all evaluated thresholds, Mel*AI*noma consistently outperforms the opticians/optometrists, suggesting that their relatively high false‐positive rate may lead to excessive referrals, while Mel*AI*noma would more efficiently minimize unwarranted referrals while still adequately identifying melanomas.

**Table 3.** Net Clinical Benefit at Various Threshold Probabilities

Threshold (%)	Opticians/Optometrists NB	Mel*AI*noma NB
10	0.1	0.2
20	0.0	0.1
30	−0.1	0.1
40	−0.3	0.1
50	−0.4	0.1
60	−0.6	0.0
70	−0.8	0.0
80	−1.2	−0.2
90	−1.6	−0.6

Threshold refers to the minimum predicted probability of melanoma at which a lesion would be referred; for example, a threshold of 30% indicates that lesions with an estimated melanoma probability of 30% or higher would be referred. NB (net clinical benefit) is defined as the proportion of true-positive referrals minus the proportion of false-positive referrals weighted by the odds at the threshold probability.

### ROC Analysis

**Figure 3.**
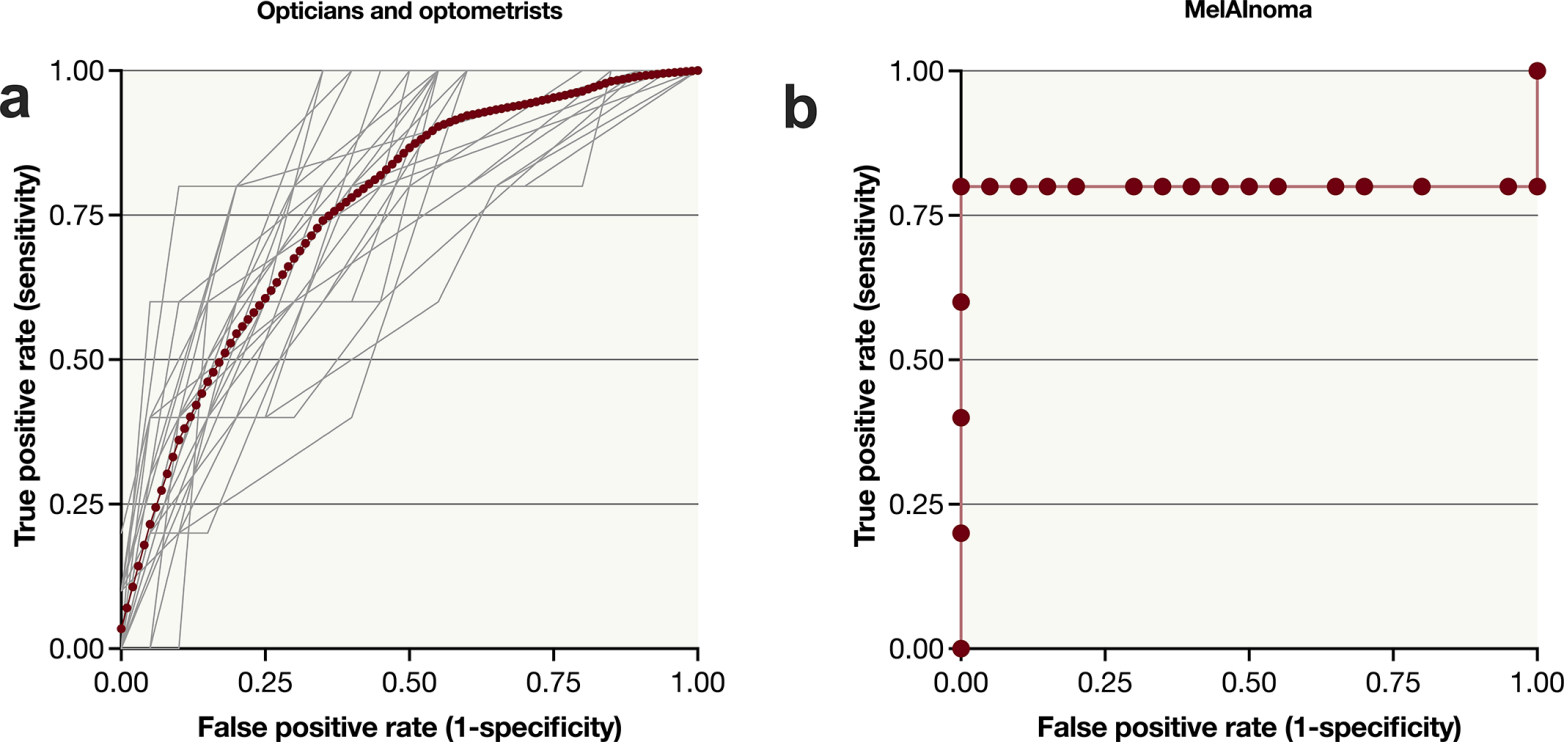
ROC curves. (**a**) ROC curves from 29 individual optometrists/opticians, each providing a MOLES score to classify 25 pigmented fundus lesions as melanoma versus nevus. The individual curves (AUC range: 0.61–0.88; mean AUC: 0.76) are depicted in *gray*, with the arithmetic mean of these curves overlaid as a *red dotted line*. (**b**) ROC curve for the Mel*AI*noma score (AUC = 0.80), which exceeded the AUCs of 22 out of 29 individual raters. DeLong's paired test comparing Mel*AI*noma versus each individual rater yielded a Bonferroni-adjusted *P* ≥ 0.99, indicating no significant differences. A paired Wilcoxon signed-rank test comparing the 29 human AUCs to the Mel*AI*noma AUC gave V = 96, *P* = 0.009.

In the ROC analysis, Mel*AI*noma achieved an AUC of 0.80 (SE = 0.18), which was numerically higher than the AUC obtained by 22 of the 29 opticians/optometrists using MOLES. The individual AUC values for opticians/optometrists ranged from 0.61 to 0.88 (mean ± SD, 0.76 ± 0.08). Paired comparisons between each optician/optometrist’s ROC curve and that of Mel*AI*noma, using DeLong's nonparametric test, yielded unadjusted *P* values ranging from 0.28 to >0.99. After Bonferroni correction for the 29 multiple comparisons, all adjusted *P* values were >0.99, indicating no statistically significant differences. However, a paired Wilcoxon signed-rank test comparing the 29 human AUCs to the Mel*AI*noma AUC indicated a statistically significant upward shift in performance for Mel*AI*noma (V = 96, Bonferroni-corrected *P* = 0.009). Notably, this test does not account for the paired ROC structure or tied values (Fig. 3).

### Optician/Optometrist Experience With Mel*AI*noma

**Figure 4.**
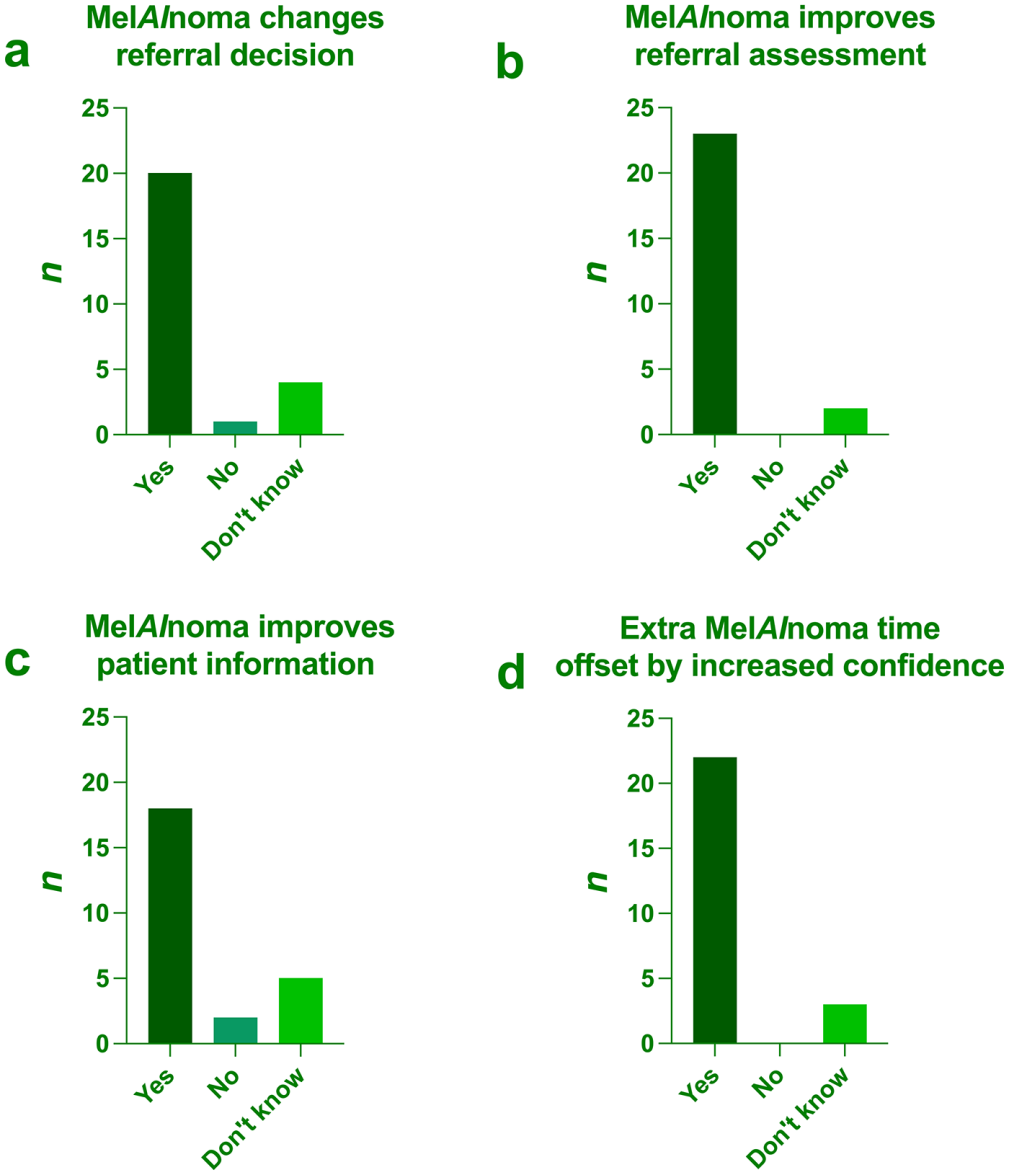
Opticians’ and optometrists’ experience after having tested Mel*AI*noma. Questions and response alternatives (in parentheses) were as follows: (**a**) According to your assessment, could an analysis with Mel*AI*noma sometimes change your evaluation of a lesion—for example, deciding that a patient should be referred to an ophthalmologist? (yes, no, don't know). (**b**) In your opinion, can Mel*AI*noma improve the assessment of which lesions should be referred to an ophthalmologist? (yes, no, don't know). (**c**) In your opinion, can Mel*AI*noma improve the information you provide to the patient? (yes, no, don't know). (**d**) Although Mel*AI*noma is not expected to lengthen assessment time and may even reduce the time required to evaluate and discuss more challenging lesions, if it did increase the time needed, would you feel that the extra time is offset by increased confidence in your assessment? (yes, no, don't know).

In the final phase of the study, opticians and optometrists were granted access to a web-based version of the Mel*AI*noma algorithm and asked to apply it to the images they had previously evaluated unaided. Upon completion, they answered a second questionnaire about their experience with the tool. Twenty-three of 25 respondents (92%) indicated that Mel*AI*noma could improve their referral decisions, while 2 (8%) were unsure. Likewise, 22 of 25 respondents (88%) agreed that any extra time required to run Mel*AI*noma would be offset by the increased confidence in their assessment; none disagreed and 3 (12%) were uncertain (Fig. 4).

## Discussion

### Main Results

In this study, we show that opticians and optometrists regularly encounter customers with pigmented fundus lesions and frequently face challenging decisions regarding referral to ophthalmologists. As anticipated, referral decisions made by opticians and optometrists showed generally high sensitivity but low specificity. Notably, diagnostic accuracy improved when decisions were guided by the Mel*AI*noma deep learning algorithm. Specifically, Mel*AI*noma outperformed opticians and optometrists by an absolute accuracy margin of 31 to 55 percentage points, provided a 4.1-fold increase in the odds of correctly referring melanomas, and offered clinical net benefit. Most of the participating professionals also expressed confidence that Mel*AI*noma would enhance their diagnostic capabilities. Given that 70% of respondents see at least five unique customers with pigmented lesions each year, integrating Mel*AI*noma into routine practice could increase their ability to confidently monitor benign lesions, thus reducing unnecessary referrals.

In a previous study using larger image data sets, Mel*AI*noma achieved a sensitivity ranging from 80% to 100% and a specificity from 74% to 81%.^11^ While individual opticians or optometrists—particularly those experienced in recognizing melanoma risk factors—may occasionally match or exceed this performance, consistent high diagnostic accuracy cannot reasonably be expected from nonspecialists. In our study, even strict adherence to guidelines resulted in relatively low specificity, with many low-risk lesions still referred to ophthalmologists. This could lead to excessive numbers of referrals and place a strain on ocular oncology clinics. In this context, AI-driven decision support tools such as Mel*AI*noma—and other emerging deep learning algorithms for pigmented-lesion triage^14^^,^^15^—could help address this challenge by improving referral precision.

Importantly, the low specificity observed here reflects guideline structure rather than clinical incompetence. Small pigmented lesions are hard to assess even for subspecialists, and MOLES functions primarily as a triage tool. Referral at MOLES ≥1 corresponds to “low-risk nevus,” so many correctly classified nevi still trigger referral.^8^ Prioritizing sensitivity—to minimize missed melanomas—inherently increases false positives when relying solely on MOLES.

Mel*AI*noma, even at the previously established cutoff of >63, achieved an 80% sensitivity and a 90% specificity. The drop from 98% (MOLES ≥1) to 80% sensitivity reflects a single false-negative among five melanomas in our small sample rather than a true algorithmic limitation. We deliberately retained the >63 threshold from our prior validation—where sensitivity and specificity were weighted equally—to avoid overfitting this limited data set. We believe it is more transparent not to alter the predefined cutoff or test multiple thresholds, which would undoubtedly yield more favorable results for Mel*AI*noma. Users can readily adjust the threshold to prioritize sensitivity at the expense of specificity or vice versa. In all cases, as shown here, Mel*AI*noma's accuracy—defined as the proportion of correctly classified lesions (true positives plus true negatives) among all cases—exceeds unaided human performance.

As research uncovers molecular factors in uveal melanoma progression—including lipid and lactate metabolism and markers of cellular quiescence^16^^–^^18^—there is growing interest in combining molecular profiling with optimized detection strategies. Future studies should prospectively validate Mel*AI*noma in larger cohorts and determine the optimal operating point for community screening: maintaining near 100% sensitivity while improving specificity enough to reduce unnecessary referrals. Incorporating Mel*AI*noma into clinical guidelines thus merits serious consideration.

### Limitations

This study has several limitations. Foremost among these is the small cohort of only 25 pigmented fundus lesion images used for assessing opticians’ and optometrists’ performance. This small number limits the strength of conclusions and implies that individual diagnostic errors significantly affect overall results. Notably, among these 25 lesions, only 5 were melanomas, meaning that a single missed melanoma reduced sensitivity from 100% to 80%.

Second, due to anonymization of respondents, we were unable to correlate individual diagnostic performances with specific responses from the initial questionnaire. Such correlations might have provided additional insights, for example, identifying factors associated with diagnostic accuracy.

Third, some statistical choices could be questioned. For example, the performance of Mel*AI*noma was evaluated using a predefined cutoff—developed with equal emphasis on sensitivity and specificity, as previously discussed—even though maximizing sensitivity is paramount in the screening setting faced by opticians and optometrists. We chose not to adapt this cutoff within the current study because, although lowering it would increase sensitivity, it would also risk overfitting the data. Likewise, one might view our use of logistic regression as merely a statistical exercise; however, it adds meaningful insight by quantifying, in a single model, the relative odds of referring a melanoma versus a nevus. The resulting odds ratio is an intuitive effect size, and the method can be extended in future work to adjust for rater-level covariates (e.g., experience or equipment) or to account for repeated measurements. Thus, logistic regression enhances interpretability, facilitates comparison of effect magnitudes across studies, and strengthens the rigor of our inference beyond simple proportion tests.

Fourth, although the gold-standard diagnoses of nevi and melanomas were established using multimodal examinations by ocular oncologists, with an accuracy comparable to histopathologic examination, exceptions do occur, particularly with small lesions.^19^ Biopsies of small lesions are uncommon, and spindle A melanocytes, often found in less aggressive uveal melanomas, are also common in choroidal nevi and normal choroidal tissue.^20^ Thus, distinguishing small, early melanomas from choroidal nevi remains challenging even when histopathologic material is available. Additionally, very few obviously benign small nevi undergo examination by ophthalmic pathologists, and those that do typically exhibit atypical features, explaining why they were biopsied initially. Finally, we did not have access to additional prognostic information (e.g., gene expression profiles, chromosome 3 status, mutations in *BAP1*, *SF3B1*, *EIF1AX* genes) or clinical features that could further characterize lesion malignancy, as all lesions diagnosed as melanomas were treated promptly with plaque brachytherapy.^18^^,^^21^^–^^24^

## Conclusions

Swedish opticians and optometrists detect small pigmented choroidal lesions with high sensitivity but poor specificity, leading to frequent, unnecessary referrals. Incorporating the Mel*AI*noma deep learning algorithm increased overall diagnostic accuracy to 88%, quadrupled the odds of correctly referring a melanoma, and reduced false-positive referrals to one-tenth of those made without the tool. Most practitioners felt that Mel*AI*noma would strengthen their assessments without prolonging consultations. Although our small image set—including only five melanomas—limits generalizability and statistical precision, these results support the potential of AI tools like Mel*AI*noma to help community eye care providers triage pigmented fundus lesions more effectively and alleviate pressure on ocular oncology clinics. Some settings may prioritize near 100% sensitivity—accepting low specificity and high referral volumes—while others may tolerate slightly lower sensitivity to curb unnecessary referrals. Health care providers, patient advocates, and policymakers should therefore determine the operating points that best fit local resources and clinical priorities.
